# Is the Most Commonly Used Strategy for the First 1,500 m of a 2,000 m Rowing Ergometer Race the Most Appropriate?

**DOI:** 10.3389/fphys.2022.827875

**Published:** 2022-03-08

**Authors:** Alice Boillet, Bastien Haas, Pierre Samozino, Baptiste Morel, Maximilien Bowen, Caroline Cohen, Laurent A. Messonnier

**Affiliations:** ^1^Laboratoire d'Hydrodynamique de l'X (LadHyX), UMR 7646 du Centre National de la Recherche Scientifique (CNRS), École Polytechnique, Palaiseau, France; ^2^Laboratoire Interuniversitaire de Biologie de la Motricité, Université Savoie Mont Blanc, Chambéry, France

**Keywords:** physiology, oxygen uptake, lactate, pacing, race, performance, human

## Abstract

This study investigated time-courses of physiological and psychological parameters of rowers during the first 1,500 m of a simulated race on a rowing ergometer using different pacing strategies. This provided a picture of the physiological and psychological state of the rowers at the start of the last 500 m of their race. Investigated strategies corresponded either to a degressive (*degr*), a progressive (*prog*), or a stable (*stab*) power output over the traveled distance. Thirteen French rowers (4 oarswomen and 9 oarsmen) of national and ex-international levels volunteered to participate. Handle force and velocity, oxygen uptake, heart rate, blood lactate concentration, and peripheral oxygen saturation were measured during the trials. Power output, generated energy [by *O*_2_ consumption (*E*_*oxi*_) and blood lactate accumulation (*E*_*non*−*oxi*_)] and efficiency were computed. Rowers also rated their perceived exertion (*RPE*) and protocol preference. In the explored strategies, no significant differences were found for *E*_*oxi*_. Final blood lactate concentration ([*La*]_*blood*_) and *RPE* were similar for all strategies. However, the increase in [*La*]_*blood*_ and *RPE* occurred sooner for *degr* than for *stab* and *prog*. Therefore, the time spent at higher [*La*]_*blood*_ and *RPE* was longer for *degr* than for *stab* and *prog*. According to the questionnaire, *degr* was the least preferred protocol. While during 2000 m races, the first 1500 m are usually and empirically often conducted in a *degr* way, the present results indicate that this strategy was the least preferred by the rowers and led to a higher time spent at high [*La*]_*blood*_ and RPE.

## 1. Introduction

The Olympic distance of rowing races is 2,000 m. The races typically last between 5'30” and 7'30” according to the boat, sex, and weight category of the rowers (World Rowing - 2020 Olympic Games Regatta, 2021). In that context, rowers use all available energetic pathways to fulfill the huge energy requirement of this type of event. Hence, elevated oxygen uptake and blood lactate accumulation have been observed during and in response to rowing races (Hagerman et al., 1978; Secher, 1993; Steinacker, 1993; de Campos Mello et al., 2009). Previous studies have shown that 70–85% and 15–30% of energy demand are provided by the oxidative and non-oxidative pathways, respectively, (Hagerman et al., 1978; Roth, 1983; Messonnier et al., 1997; Russell et al., 1998; de Campos Mello et al., 2009).

During races, rowers adopt a strategy that can be retrospectively investigated from the time-course of speed (Garland, 2005). Like in any other racing sport, the presence of opponents changes the strategy (Hettinga et al., 2017). Rowers partly adjust/adapt the speed of the boat, their performance and their pacing strategy (Edwards et al., 2016) to the stakes of the race (qualifications or finals) (Chu et al., 2021). Muehlbauer et al. (2010) and Chu et al. (2021) found that the most observed strategy at the international level corresponds to a parabolic profile of speed: fast start in the first 500 m, slight deceleration in the second and third 500 m, and final acceleration in the last 500 m [keeping in mind that power output increases non-linearly with the velocity (with the cube of the velocity at constant speed)]. However, it is not known whether this strategy corresponds to an optimum in terms of psychological and/or energy management. Studying 228 crews of the Boat Race (Oxford-Cambridge), Edwards et al. (2016) observed that 81% of the teams in the lead after the first quartile of the race won the duel. Whether the winners were advantaged (i) by their leadership position, allowing them to adapt to variations in pace of their opponents, (ii) by their greater physical abilities and their management during the race, or (iii) by a combination of these possibilities, the question remains open. Note that the distance of the Boat Race (6,800 m) is not the Olympic distance. Contrary to this strategy of a fast first 500 m, some rowers adopt a more constant pacing strategy. If with this strategy, the rowers are not first at the beginning of the race and do not benefit from the potential advantage of being in the lead (Murray et al., 2016) and having a visual on their opponents, they may still manage to win the race. Although the velocity pattern was still U-shaped (Garland, 2005), these crews raced with speed changes of less amplitude. These findings put forward the question whether a more even pacing strategy, or a progressive strategy, is associated with higher performance in rowing races and should be considered by elite rowers and their coaches. The literature on middle-distance events (3–7 min) in other sports is not consensual either on this point. If certain retrospective analyzes (Foster et al., 1993) or optimization models (van Ingen Schenau et al., 1990) tend to show that a more even pacing strategy would be more efficient to achieve better performance, other studies, observing that winners were those whose speed varied the most in races (Mytton et al., 2015; Taylor et al., 2016), do not confirm this standpoint.

The present study aimed to investigate the individual physiological and psychological responses of rowers to different pacing strategies over the first 1,500 m of a race. This investigation allowed to assess the physiological and psychological state the rowers at each step of the 1,500 m and especially at the start the final and determinant 500 m. The initial hypothesis is that a non-degressive effort could be more optimal from a physiological and psychological point of view.

## 2. Methods

### 2.1. Subjects

Thirteen French rowers of national and ex-international levels volunteered to participate in the study. The group included 4 oarswomen and 9 oarsmen. Their height, age, and weight were (mean ± SD) 178±9 cm, 22±4 yo, and 73±10 kg, respectively. The rowers had 10±4 years of experience in rowing and performed 6±2 training sessions per week. Although less marked in women (Chu et al., 2021), both sexes adopt on average a parabolic (U-shaped) curve of speed during the races (Garland, 2005; Chu et al., 2021). This gives support to include both sexes in the same study. The experiments were based on the 2000 m time trial performed within the same rowing season. The study was approved by the local ethics committee and conducted in agreement with the declaration of Helsinki. Before giving their written informed consent, subjects were advised of the objectives, all risks, possible discomforts, and potential benefits of the experiment.

### 2.2. Protocols

All tests were conducted on a wind resistance braked rowing ergometer (Concept II model C, Morrisville, VT, USA). Performing the experiments on a stationary ergometer facilitated monitoring of the different measured parameters. The participants were accustomed to the use of the apparatus. The computer of the ergometer continuously displayed power output, stroke rate and distance traveled.

**Figure 1:**
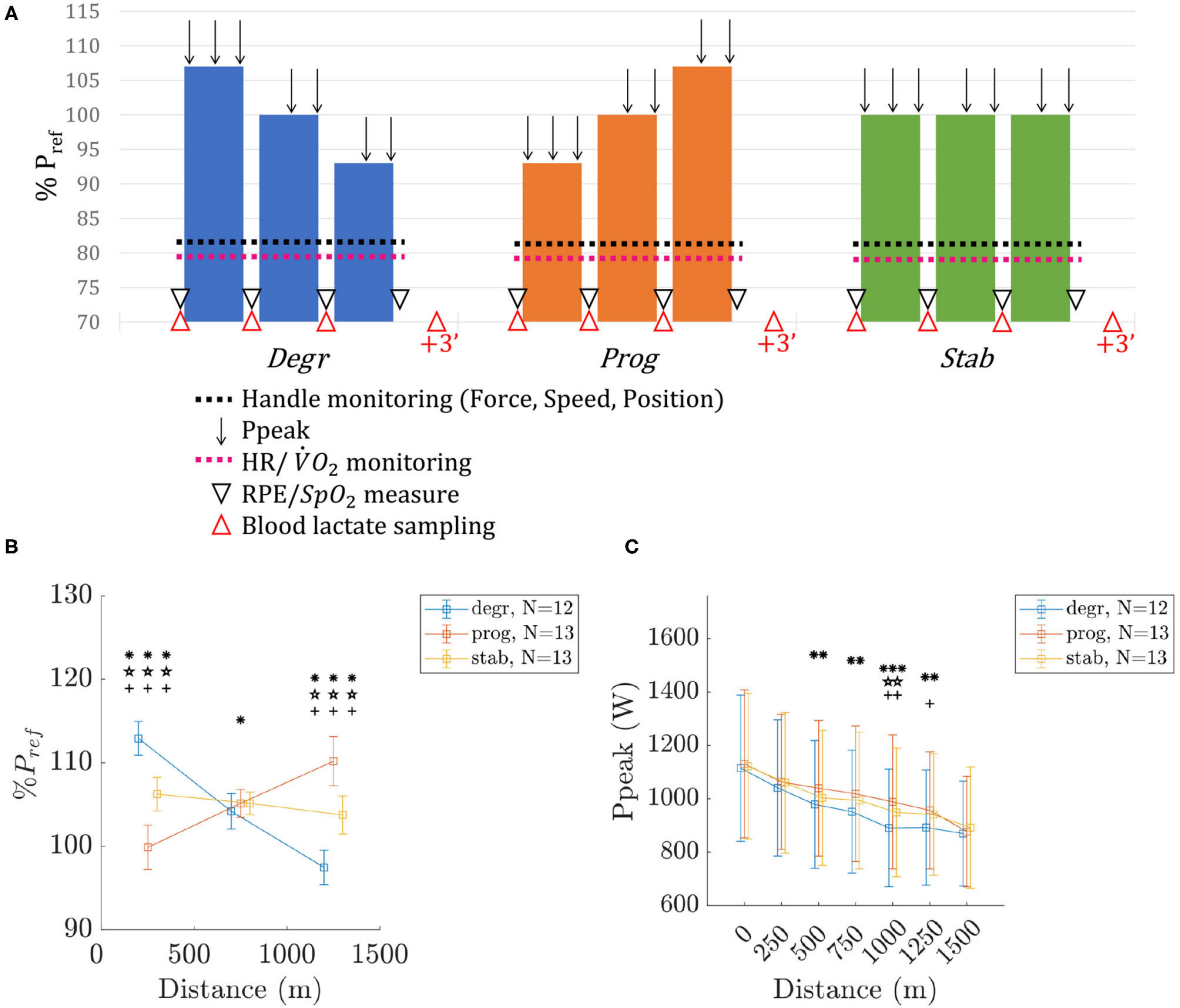
Protocol design **(A)**, mean power on each 500 m **(B)** and *Ppeak* time-courses **(C)**. Values are mean ± SD. The symbols *, ⋆, and + indicate a significant difference between *degr*-*prog, prog*-*stab*, and *stab*-*degr*, respectively. The number of symbols (one, two, or three) indicate the *p*-value (<0.05, <0.01, and <0.001, respectively).

Each athlete performed three trials, except for one who performed only two because of an injury. A summary of the protocols and measured parameters is available in Figure 1A. The protocol consisted in performing a 1,500 m on rowing ergometer in three different conditions at least 48 h apart (excepted once where only 24 h separated two consecutive trials). The three visits were carried out as far as possible, at a similar time of the day, in the same prandial state, and in the same energetic status (diet). The order in which the conditions were carried out was randomly assigned.

Each trial consisted of performing 1,500 m on rowing ergometer divided in three 500 m with a degressive (*degr*), progressive (*prog*), and stable (*stab*) profile/design in terms of mechanical power output performed during the test. Every 500 m was associated with a target power output determined as follows for each athlete and strategy:

For *degr* : *P*_500_ = 107*%P*_*ref*_; *P*_1000_ = *P*_*ref*_; *P*_1500_ = 93*%P*_*ref*_.
For *prog* : *P*_500_ = 93*%P*_*ref*_; *P*_1000_ = *P*_*ref*_; *P*_1500_ = 107*%P*_*ref*_.
For *stab* : *P*_500_ = *P*_1000_ = *P*_1500_ = *P*_*ref*_.

where *P*_*ref*_ = 97.5% of the mean power output sustained during the best 2,000 m all-out performance test of the season. *P*_500_, *P*_1000_, and *P*_1500_ correspond to the target power output of the first, second and third 500 m.

At the beginning of each visit, a hyperemic cream was applied to the earlobe for arterialization of the capillary blood. The athletes then performed a self-paced warm-up on land (flexibility), on a cycling ergometer, on a rowing ergometer, or a combination of these possibilities. On rowing ergometer, the warm-up included accelerations and peak strokes. The rowers self-selected their drag factor (identical drag factor for all tests/visits for a given athlete). Subjects were then equipped with the measuring instruments for the following parameters: gas exchanges, heart rate and peripheral oxygen saturation (see Supplementary Materials for details). The hyperemic cream was removed from their earlobe and a blood lactate sample was taken to measure the pre-trial blood lactate concentration.

For the first 500 m, subjects were instructed to reach the target power output (typically in 3–5 strokes) as quickly as possible. Once stabilized at the target power output, the rowers had to perform one maximal stroke (typically the fifth or sixth stroke) to determine *Ppeak*. Then rowers turned back to the target power. Right at 250 m and just before reaching 500 m, the athletes performed a maximal stroke to determine *Ppeak*, then returning to the target power output or stopped, respectively. The same procedure was repeated thereafter for the second and third 500 m. A total of 7 *Ppeak* were thus performed during the 1,500 m trial. A rest period between two consecutive 500 m exercise bouts allowed to take a blood lactate sample at the earlobe and the recording of the rate of perceived exertion (RPE, 1–10 scale). The duration of the rest periods was strictly limited to the time necessary for the measurements (15±4 s). A post-trial blood lactate sample was taken 3 min after exercise completion.

### 2.3. Measurements

Detailed protocols for the measurements of the mechanical (force, velocity, and power output), physiological [oxygen uptake ($\dot{V}O_{2}$), time-constant of the $\dot{V}O_{2}$ response during the first 500 m (τ), $\dot{V}O_{2}$ at steady state ($\dot{V}O_{2}SS$), oxidative contribution to energy supply (*E*_*oxi*_), heart rate (HR), heart rate at steady state (HRSS), blood lactate concentration ([*La*]_*blood*_), non-oxidative glycolytic contribution to energy supply (*E*_*non*−*oxi*_), lactate area under the curve (*AUC*_*La*_), peripheral oxygen saturation (*SpO*_2_), and efficiency (ϵ)] and psychological [rating of perceived exertion (RPE), RPE area under the curve (*AUC*_*RPE*_) and grading of protocols by athletes] parameters are available in Supplementary Materials.

### 2.4. Statistical Analysis

Trials for which the measuring device was defective were removed from the statistical analyses. The size of each data group will be specified (*N*). The subject who performed only two of the three conditions was included in the one-to-one comparisons of the two conducted trials. All data processing and statistical analyses were performed on MATLAB. Means (± standard deviation) were calculated by standard methods. On the graphs, the solid squares represent the means and the error bars represent the standard deviations. In the box plots, the segments delimit the first quartile, the median and, the third quartile. The hollow circles correspond to the individual results. The Kolmogorov–Smirnov test was used to refute the assumption of normality. The paired-Wilcoxon rank test was then used to compare the groups two by two. The statistical significance threshold was set at p-value <0.05. On the figures, the symbols *, ⋆, and + indicate a significant difference between *degr*-*prog, prog*-*stab*, and *stab*-*degr*, respectively. The number of symbols (one, two, or three) indicate the *p*-value (<0.05, <0.01, and <0.001, respectively). The effect size was evaluated through a rank-biserial correlation. Numerical values of *p*-values and rank-biserial correlation are available in the Supplementary Materials.

## 3. Results

### 3.1. Power Outputs

The mean power output for each 500 m and the different protocols are available in Figure 1B. The average variations between two consecutive 500 m bouts were −7.1±0.1%, +5.1±0.2%, −1.2±0.1% for *degr, prog, stab*, respectively. Although the variation for *prog* was below the targeted value (+7%), there still was a significant difference when compared to the other protocols and therefore the results can be analyzed considering that the protocols have been respected. In accordance, time duration to complete the 1,500 m trials (removing the stopping times for data collection) were similar among trials: 305±31 s, 304±29 s, 304±31 s for *degr, prog*, and *stab*, respectively.

The time-courses of *Ppeak* are available in Figure 1C. Initial and final *Ppeak* did not differ significantly between protocols. However, *Ppeak* in *degr* was lower than in *prog* between 500 and 1,250 m and than in *stab* between 1,000 and 1,250 m. *Ppeak* in *stab* was also lower at 1,000 m than *Ppeak* in *prog*.

### 3.2. Physiological Measurements

#### 3.2.1. Oxygen Uptake

**Figure 2:**
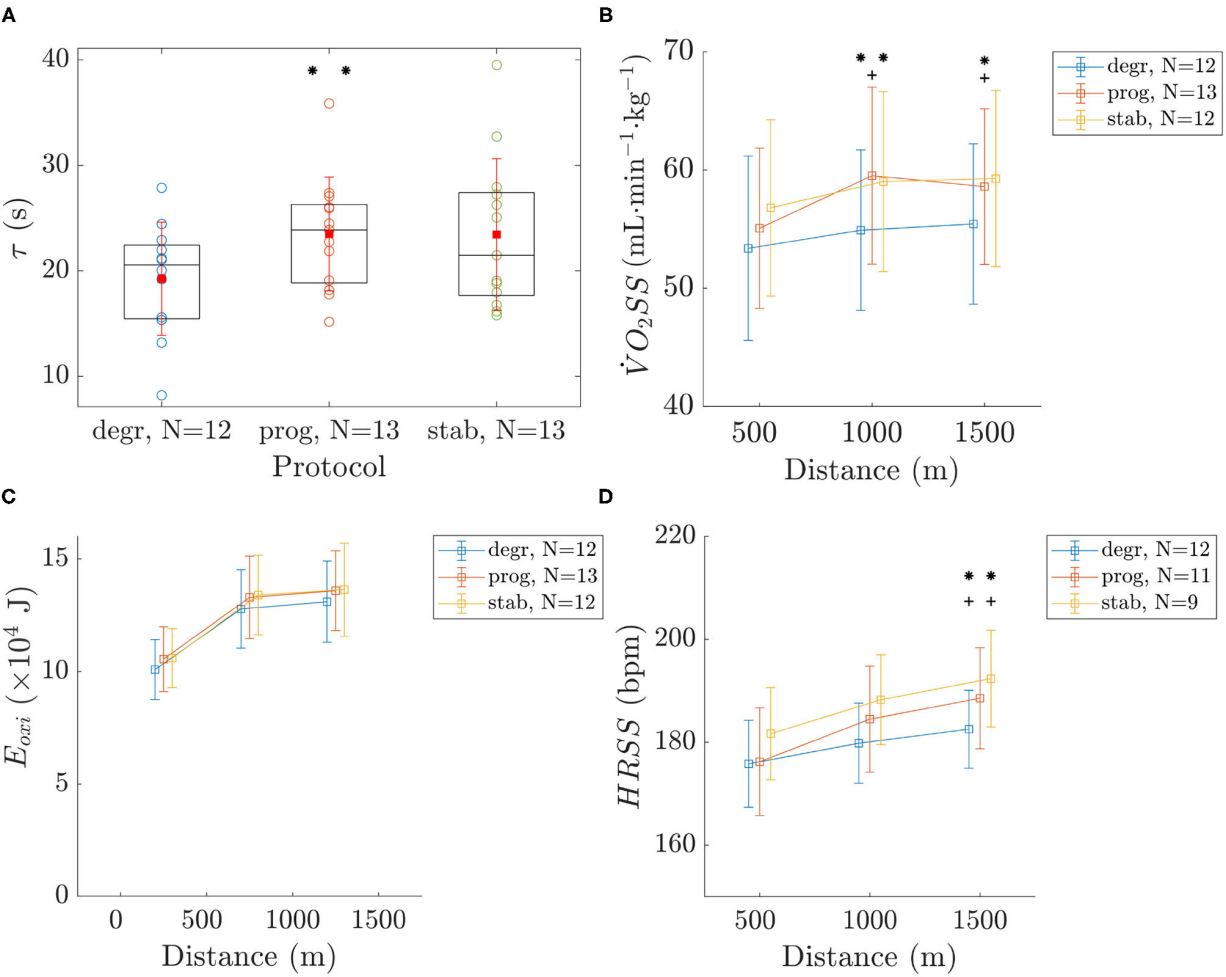
τ (time constant of the primary response of the oxygen uptake on the first 500 m) **(A)**, $\dot{V}O_{2}SS$ **(B)**, *E*_*oxi*_ **(C)**, and *HRSS* **(D)**. Values are mean ± SD. The symbols * and + indicate a significant difference between *degr*-*prog* and *stab*-*degr*, respectively. The number of symbols (one or two) indicate the *p*-value (<0.05 and <0.01, respectively).

Results drawn from $\dot{V}O_{2}$ are reported in Figure 2. The time constant τ (Figure 2A) was significantly lower in *degr* than in *prog*. τ in *stab* did not significantly differ from the other conditions (Figure 2A). $\dot{V}O_{2}SS$ was lower in the 2nd and 3rd 500 m for *degr* than in *stab* and *prog* (Figure 2B). *E*_*oxi*_ for each 500 m bout was not different between conditions (Figure 2C).

#### 3.2.2. Heart Rate

The *HRSS* (Figure 2D) was lower in the last 500 m for *degr* than in *stab* and *prog*.

#### 3.2.3. Blood Lactate Concentrations

**Figure 3:**
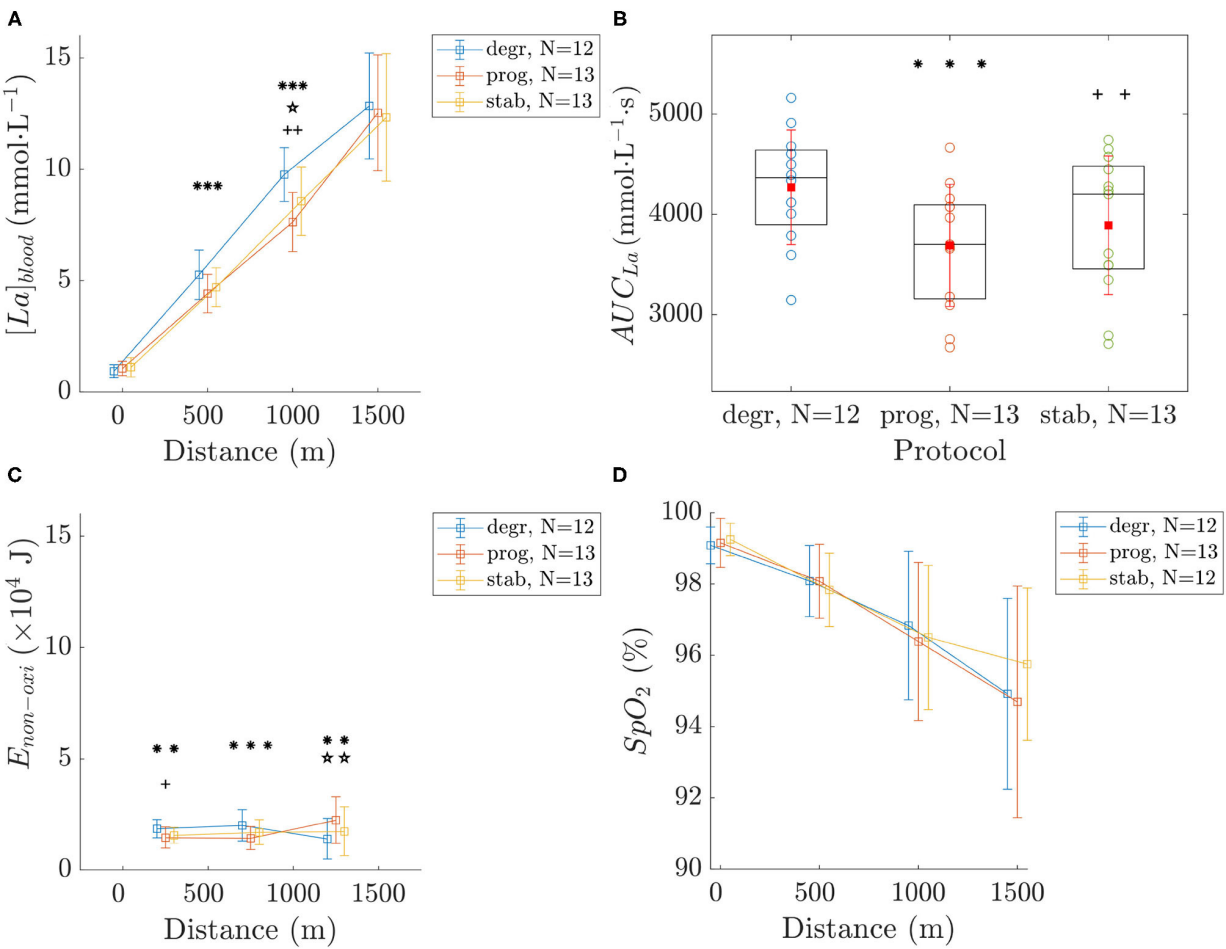
[*La*]_*blood*_ **(A)**, *AUC*_*La*_ **(B)**, *E*_*non*−*oxi*_ **(C)**, and *SpO*_2_ **(D)**. Values are mean ± SD. The symbols *, ⋆, and + indicate a significant difference between *degr*-*prog, prog*-*stab*, and *stab*-*degr*, respectively. The number of symbols (one, two, or three) indicate the *p*-value (<0.05, <0.01, and <0.001, respectively).

Results drawn from [*La*]_*blood*_ are reported in Figure 3. Initial and final values of [*La*]_*blood*_ were similar for all conditions (Figure 3A). However, after the first 500 m, [*La*]_*blood*_ was higher for *degr* than for *stab* and *prog*, while no difference was observed between the two latter conditions. At 1,000 m, [*La*]_*blood*_ were higher in *degr*, lower in *prog* and in between in *stab*.

*AUC*_*La*_ (Figure 3B) was significantly greater for *degr* than for the two other conditions. A higher *AUC*_*La*_ corresponds to a longer time spent at higher [*La*]_*blood*_.

*E*_*non*−*oxi*_ (Figure 3C) was higher on the first 500 m for *degr* than for *prog* and *stab*. On the second 500 m, *E*_*non*−*oxi*_ was higher for *degr* than for *prog* with no significant difference with *stab*. Finally, for the last 500 m bout including the first 3 min of recovery, *E*_*non*−*oxi*_ was greater for *prog* than for the other conditions.

#### 3.2.4. Blood Oxygen Saturation

No significant differences were observed in time-courses of *SpO*_2_ between the different conditions (Figure 3D).

#### 3.2.5. Efficiency

Efficiencies were 25 ± 8%, 25 ± 8%, and 26 ± 2% for *degr, stab*, and *prog*, respectively. These values are coherent with the literature (Fukunaga et al., 1986).

### 3.3. Psychological Variables

**Figure 4:**
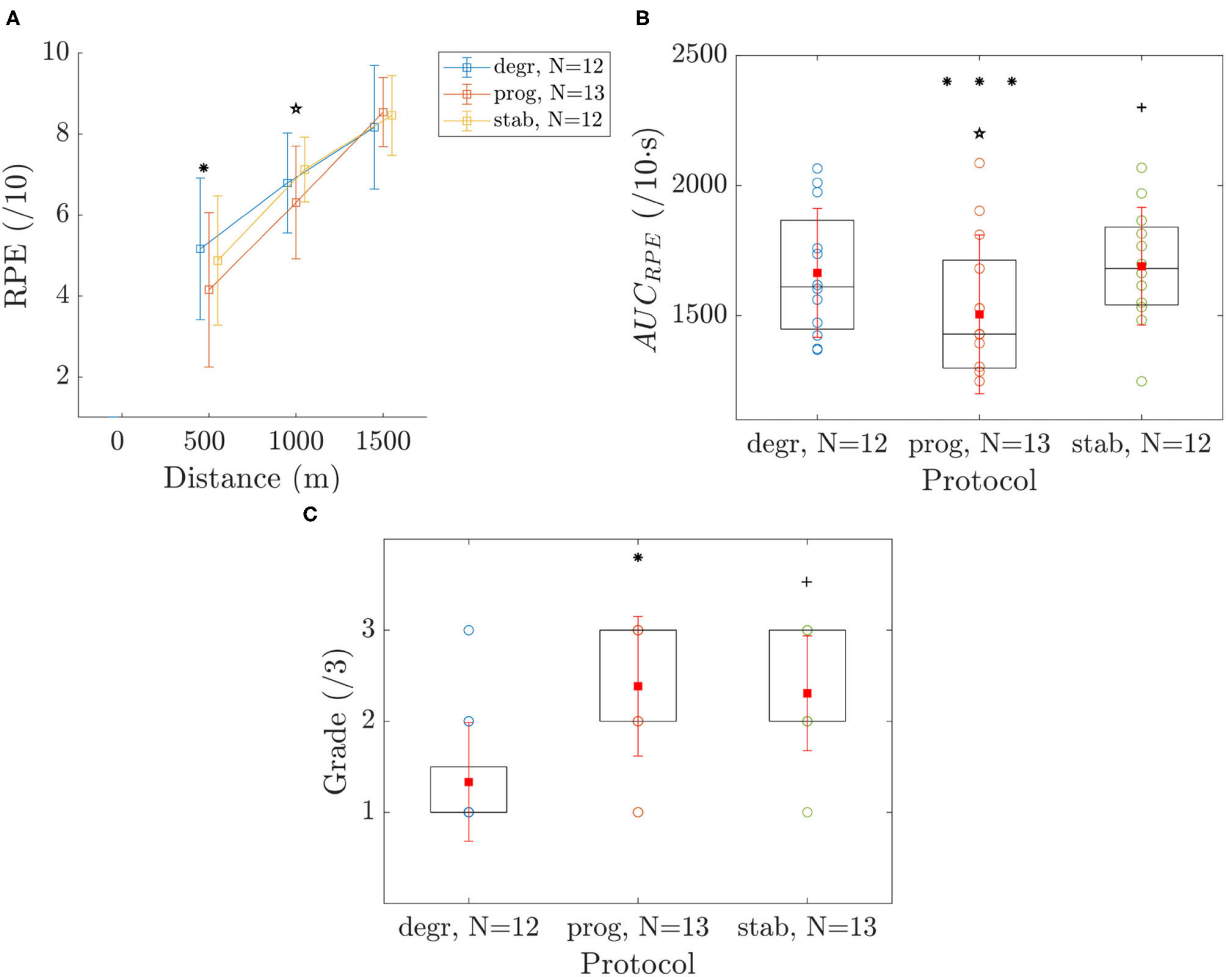
RPE **(A)**, *AUC*_*RPE*_ **(B)**, and grading of protocol **(C)**. Values are mean ± SD. The symbols *, ⋆, and + indicate a significant difference between *degr*-*prog, prog*-*stab*, and *stab*-*degr*, respectively. The number of symbols (one, two, or three) indicate the *p*-value (<0.05, <0.01, and <0.001, respectively).

RPE (Figure 4A) was similar at the end of the 1,500 m trials in the three conditions. However, RPE was lower in *prog* than in *degr* at 500 m and than in *stab* at 1000 m.

*AUC*_*RPE*_ (Figure 4B) was significantly greater for *degr* than for the two other conditions. *AUC*_*RPE*_ also was greater for *stab* than for *prog*.

The grading of conditions by athletes (Figure 4C), reported that *stab* and *prog* conditions were preferred than *degr*, with no difference between the two first.

## 4. Discussion

This study aimed to investigate the physiological and psychological responses to three pacing strategies on the first 1,500 m of a rowing race. Research has already been done in imposing a strategy to rowers on ergometer (Lander et al., 2009) but not in the range of power developed by the athletes during 2000 m races. The three strategies used in the present study (*degr, stab*, and *prog*) were equivalent in terms of performance, meaning that power output, time to travel the distance and thus work were similar in the three conditions. Contrary to our initial hypothesis, most parameters of interest measured during the 1,500 m trials (*Ppeak*, [*La*]_*blood*_ and RPE) were not different at exercise completion between the three strategies. On the other hand, time-courses of several parameters were different between trials so that *Ppeak* was lower and [*La*]_*blood*_ and RPE were higher in *degr* compared to *stab* and *prog* during and after the second 500 m. Finally, athletes clearly disliked the *degr* protocol.

### 4.1. Muscle Function

From a mechanical point of view, the 1,500 m trials were tiring (as illustrated by the decrease of *Ppeak* throughout the trials) but not in the same way according to the trials. Indeed, while initial and final *Ppeak* were similar, *Ppeak* in *degr* was lower than in *prog* between 500 and 1,250 m and than in *stab* between 1,000 and 1,250 m. It is interesting to note, that the difference between the trials are the most apparent on the middle 500 m while this is the period where the power output was similar between trials. This suggests that muscle function at 1,000 m is still under the dependence of what has been done during the first 500 m (*vide infra*).

### 4.2. Physiological Responses

Pacing strategies affected physiological responses. Interestingly, and similarly to what has already been described for muscle function, the discrepancies between the strategies were most often apparent during and after the second 500 m, while the power output was similar in the different conditions.

The first surprising result was that during the second 500 m, $\dot{V}O_{2}SS$ was lower in *degr* than in *stab* and *prog* while the power output were the same in the three conditions. There is very few probabilities that this result is a type I error insofar as $\dot{V}O_{2}SS$ during the first 500 m in *degr* was also significantly (p < 0.05) lower than $\dot{V}O_{2}SS$ during the third 500 m in *prog* (data not shown). The present study does not allow to find a definite explanation for this result. However, taking into account (i) that the lower $\dot{V}O_{2}SS$ in *degr* were accompanied by higher [*La*]_*blood*_, (ii) that higher [*La*]_*blood*_ are associated with lower pH (acidosis) (Stewart, 1983), and (iii) that acidosis inhibits oxidative phosphorylation *in vivo* (Jubrias et al., 2003), one cannot exclude that the lower $\dot{V}O_{2}SS$ observed in the present study at 1,000 m is the result of a lower muscle pH. However, further studies are necessary to confirm/infirm this speculation/hypothesis.

Taken into account τ and $\dot{V}O_{2}SS$, it resulted that oxidative energies were similar among conditions. These results are similar to those obtained by Hettinga et al. (2007) on 1,500 m cycling time trials conducted with three strategies similar to the ones used here, suggesting that pacing strategies have no significant impacts on the overall contribution of oxidative metabolism in energy supply.

During the first 500 m, [*La*]_*blood*_ increased faster in *degr* than in *prog*. This result indicates that the higher power output was not compensated by the faster increase in $\dot{V}O_{2}$ (i.e., the lower τ) in *degr*, resulting in higher [*La*]_*blood*_. The increase of the differences in [*La*]_*blood*_ between the conditions after the second 500 m, while the power outputs were similar in the three conditions, may be explained by the delay of muscle lactate to reach the blood. At that point, it is tempting to link the time-course of [*La*]_*blood*_ with those of *Ppeak* since *Ppeak* curves mirror those of [*La*]_*blood*_. In a previous study, Hogan et al. (1995) observed that an increase in [*La*]_*blood*_ induced a decrease of force production (muscle studied *in situ*). However, a causative link between elevated [*La*]_*blood*_ and low *Ppeak* is highly speculative, highly controversial, and unlikely in the present case.

In the present study, *AUC*_*La*_ was higher in *degr* than in *stab*, this later being itself higher than in *prog*. This latter result illustrates that the athletes spent a longer time at higher [*La*]_*blood*_ in *degr* than in *prog* and *stab*.

### 4.3. Psychological Responses

Results for RPE were reminiscent with those for [*La*]_*blood*_. This is consistent with the association between [*La*]_*blood*_ and the perception of an effort (Borg et al., 1987). Moreover, *AUC*_*RPE*_ was higher in *degr* than in *prog* and *stab*, indicating that athletes spent a longer time at higher RPE in *degr* than in the two other conditions.

If it is difficult to infer whether there is a link between the RPE profile during the trials and the rating of conditions by the athletes, one may nevertheless report that for the athletes, the worse strategy was *degr*. At least, *degr* was the strategy they disliked the most. This result is relatively surprising since this is the most often strategy observed during the first 1,500 m of races (Garland, 2005; Muehlbauer et al., 2010; Chu et al., 2021). Four possible explanations may account for the discrepancy between the personal rating of the rowers and what they actually do during races, and one cannot exclude that the explanation lies in a combination of the following possibilities.

First, starting the race in the lead offers a certain psychological advantage. This situation gives (i) athletes confidence and (ii) a visual control on their opponents, allowing them to adapt and modulate their effort according to possible catching up of the other competitors. The poor rating of the *degr* strategy in the present study might then be explained by the absence of opposition and visual on this opposition.

Second, *degr* strategy could be advantageous for the boat speed from a mechanical point of view. This could be investigated through a mechanical model taking into account (i) the propulsive power generated by the athletes and (ii) the resistive properties of the (considered) boat. One of the resistive terms, namely the added mass, is proportional to the acceleration of the boat (Cabrera et al., 2006; Formaggia et al., 2009). Therefore, in a *degr* strategy, the initial added mass term would be of great magnitude at the beginning of the race. During the following deceleration, this added mass term would result in a propulsive contribution for the boat. However, such a model needs to be further investigated in order to determine and quantify the benefit, if it exists.

Third, there may be a physiological reason to perform higher power outputs at the beginning. As fatigue develops during the race, it may become more and more difficult to perform high power outputs. In other words, the high level of power outputs, generated in the early phase of a race in a *degr* scenario, might be impossible to produce later, especially during the last 500 m of a race, even if a more “energy-sparing” strategy (e.g., *prog*) was used at the beginning of the race. In accordance with this possibility, one may note that in the present study, the mean increase in power output in (*prog*) was of only +5.1 ± 0.2% instead of the required 7%. Indeed, it was difficult for some athletes to increase the power output in the final 500 m of the *prog* condition.

Last, this strategy may be the simplest for the athletes, insofar as the other strategies (*prog* or *stab*) would require rowers to know precisely their physical abilities and their energy reserve at any time of the race. Therefore, starting stronger and letting the deceleration be dictated by the occurrence of fatigue may ensure the athletes to provide a maximum effort, whereas starting with less power output could result in finishing the race without having used all energetic reserves. In favor of this last argument, Hoffmann et al. (2014) have shown that energy management on a 2000 m ergometer race was improved in the presence of a “rowing avatar” that indicated them the pace to follow during the simulated race.

To conclude, a contradiction exists between athletes' preferences and field observations. To understand this contradiction, it would be interesting/necessary to include a “performance” trial in a future study. Indeed, it would be of great interest to quantify the maximum effort achievable in each strategy configuration to be able to conclude on the most optimal strategy (*vide infra* 4.4 for additional words in that regards).

### 4.4. Limitations and Perspectives

We are aware that this study has limitations. One first criticism may lie on the fact that rowers were stopped at each 500 m. Because we intended to measure time-course of parameters during the different trials and because it is highly difficult to perform some measurements during physical activity (e.g., [*La*]_*blood*_), we choose to stop the athletes every 500 m. A second criticism may lie on the fact that athletes did not perform a complete 2000 m. This choice is justified by several aspects. First of all, none of the athletes accepted to perform three all-out 2,000 m rowing exercises. Second, the type of effort required in the present study was not usual for the rowers. They had to follow strategies they do not necessarily apply during races. Most of the rowers of the present study would have been unable to travel an extra 500 m. Retrospectively, not having pushed the distance to 2,000 m was reasonable since some athletes were in trouble or even did not succeed to respect precisely the imposed power outputs, attesting of the exhaustion of the rowers. Along the same line, one may note that [*La*]_*blood*_ measured at the end of the 1,500 m trials were approaching the levels found after a 2,000 m race (Hagerman et al., 1978; Nielsen et al., 2002). Another limitation is that under none of the conditions performed in the present study did the rowers complete a self-paced 1,500 m. An interesting study (Lander et al., 2009) suggested that a self-paced trial might have been a more effective strategy than any of the other conditions proposed. Therefore, it cannot be excluded that the crews that adopt a more even (but still U-shaped) pace and win the race are those that are able to ignore their opponents and to race at their own pace. Despite the limitations mentioned above, we believe that our results are still of great interest in understanding the influence of pacing strategy on a 2,000 m rowing event. Our results underline the intra-trial differences in the time-course of psychological and physiological variables for different strategies on the first 1,500 m of a race.

## 5. Conclusion

Contrary to our initial hypothesis, While *Ppeak*, *E*_*oxi*_, [*La*]_*blood*_ and RPE were similar at the start and at the end of the different conditions, time-courses of several of these parameters were different. It resulted that (i) *Ppeak* was lower and (ii) [*La*]_*blood*_ and RPE were higher in *degr* than in *prog* and *stab* especially in the middle 500 m while the power outputs were the same between trials. These results indicate (i) that the alterations of muscle function and physiological responses are delayed compared to the actual power output performed and (ii) that determination of the time-course of parameters during exercise brings more insights than just pre- and post-exercise measurements. Athletes retrospectively disliked the *degr* strategy the most, which is surprising since it is the one to most often used in competition during the first 1,500 m. This contradiction might be explained by the fact that using a degr strategy could be more efficient in terms of performance either from a positioning with respect to competitors, mechanical, physiologically achievable effort, or tactical implementation. Further studies are necessary.

## Data Availability Statement

The raw data supporting the conclusions of this article will be made available by the authors, without undue reservation.

## Ethics Statement

The studies involving human participants were reviewed and approved by Comité d'Éthique de la Recherche de l'Université Savoie Mont Blanc. The patients/participants provided their written informed consent to participate in this study.

## Author Contributions

LM, PS, CC, and BM designed and conducted this study. MB was in charge of the experimental set-up design. LM, BH, MB, CC, and AB conducted the experiments. AB and LM wrote the first draft of the article and analyzed data. All authors critically reviewed the draft and approved the final version.

## Funding

This work was funded by a grant from the PIA (Programme d'Investissements d'Avenir) via the ANR (Agence Nationale de la Recherche) with the reference ANR-20-STHP-0006.

## Conflict of Interest

The authors declare that the research was conducted in the absence of any commercial or financial relationships that could be construed as a potential conflict of interest.

## Publisher's Note

All claims expressed in this article are solely those of the authors and do not necessarily represent those of their affiliated organizations, or those of the publisher, the editors and the reviewers. Any product that may be evaluated in this article, or claim that may be made by its manufacturer, is not guaranteed or endorsed by the publisher.
