# Supplementary material for: Is the Most Commonly Used Strategy for the First 1,500 m of a 2,000 m Rowing Ergometer Race the Most Appropriate?
Source: Front Physiol. 2022 Mar 8;13:827875. doi: 10.3389/fphys.2022.827875 (PMC8958042; doi:10.3389/fphys.2022.827875)
Supplement: Supplementary file 1 [file Presentation_1.PDF]

Supplementary Material

1 MEASUREMENT PROTOCOLS

1.1 Power measurements

Force at the handle was continuously recorded at 200 Hz with an analogic force sensor (Model 615, Tedea, Huntleigh, Vishay, CA) screwed in between the chain and the handle. Horizontal position and resulting velocity were recorded thanks to an optical sensor located at the inertial wheel. Synchronized measurements of horizontal velocity and force allowed the computation of effective power output exerted by the athlete at the handle. The computed power output (P_{handle}) was higher than the one displayed by the Concept II ergometer (by $\approx 12\%$). The discrepancy is due to the calculus method used by the latter (Boyas et al., 2006). However, this did not jeopardized the interpretations of the data. For $Ppeak$, the power is averaged over the drive phase, identified by a positive force (in the pulling direction) exerted at the handle.

1.2 Physiological measurements

1.2.1 Oxygen uptake

Oxygen uptake ($\dot{V}O_2$) was continuously monitored during the 1500 m trial using a calibrated metabolic cart (Cardiorespiratory Diagnostic System, ULTIMA PFX, MGC Diagnostics Corp., USA). $\dot{V}O_2$ was recorded at 1 Hz and expressed in mL·min^{-1}·kg^{-1}.

Ten seconds after the beginning of the first 500 m, the $\dot{V}O_2$ signal was fitted using an iterative least-squares method by the function:

$$\dot{V}O_2(t) = A(1 - e^{-t/\tau}) \tag{S1}$$

where A is a constant denoting the amplitude of the exponential term (mL·min^{-1}·kg^{-1}) and τ is the characteristic time-constant of the primary response of the oxygen uptake as defined by Ingham et al. (2007). The 10 seconds interval was applied to avoid disturbance due to the cardio-dynamic component as described by Ingham et al. (2007).

Oxygen uptake at steady state ($\dot{V}O_2SS$) was determined as the mean $\dot{V}O_2$ over the last 15 seconds of each 500 m.

O_2 uptake was considered as a measure of oxidative energy pathway contribution (Di Prampero, 1981). Therefore, on a given time interval $[t_0, t_f]$ the $\dot{V}O_2$ dynamic can be roughly converted into amounts of energy produced (E_{oxi}).

$$E_{oxi} = \int_{t_0}^{t_f} \dot{V}O_2(t) C_1 \, dt \tag{S2}$$

With $\dot{V}O_2$ expressed in mL·s^{-1}. $C_1(= 20.1$ J·mL$^{-1})$ is the conversion factor of O_2 (mL) into energy (J) (Di Prampero, 1981).

1.2.2 Heart Rate

Heart rate (HR) was continuously recorded during the 1500 m at 1 Hz with a heart-rate sensor coupled to a heart rate monitor (Polar M430, Polar Electro Oy, Finland) and expressed in beats per minute (bpm). On each 500 m, HR at steady state ($HRSS$) was similarly determined as $\dot{V}O_2SS$.

1.2.3 Blood Lactate concentration

Twenty μL of arterialized capillary blood was sampled from the earlobe before the 1500 m trial, between two consecutive 500 m bouts and 3 minutes after the end of exercise. The 3-min delay after exercise completion corresponds to the time necessary to balance muscle and blood lactate concentrations (transit time between muscle and blood) and therefore to reach the peak $[La]_{blood}$ after the 1500 m (Messonnier et al., 1997). Blood was immediately placed in Eppendorf containing saponin (for hemolysis) and 180 μL of a dilution buffer (Geyssant et al., 1985). Tubes were then stored at 4 °C until analysis. Blood lactate concentration ($[La]_{blood}$) was determined enzymatically in whole blood using a YSI 2300 STAT Plus analyzer (YSI Inc., Yellow Springs, OH, USA). Lactate area under the curve (AUC_{La}) was computed for each condition as (since the sampling frequency is very low) the following:

$$AUC_{La,prot} = \sum_{i=1}^{3} \frac{[La]_{blood,i-1} + [La]_{blood,i}}{2} \cdot (t_i - t_{i-1}) \tag{S3}$$

where $[La]_{blood,i-1}$ and $[La]_{blood,i}$ were the blood lactate concentration before and after the ith 500 m of the trial, respectively, $(t_i - t_{i-1})$ being the duration of the ith 500 m.

Blood lactate accumulation was considered as a measure of non-oxidative glycolytic energy pathway contribution (Di Prampero, 1981). Therefore, on a given time interval $[t_0, t_f]$, $[La]_{blood}$ increase can be converted into amount of energy produced ($E_{non-oxi}$).

$$E_{non-oxi} = ([La]_{blood}(t_f) - [La]_{blood}(t_0))C_1C_2 \cdot BM \tag{S4}$$

where BM is the body mass of the considered athlete in kg, C_2 is the oxygen equivalent of lactate i.e., 3 mL $O_2 \cdot$ kg $^{-1}$ per mmol $\cdot$ L^{-1} of blood lactate accumulation (Di Prampero, 1981). Note that for the final 500 m, $[La]_{blood}(t_f)$ is taken 3 minutes after exercise completion (time needed to balance muscle and blood lactate concentrations).

1.2.4 Peripheral oxygen saturation

Peripheral oxygen saturation (SpO_2, %) was continuously measured at the earlobe (Nonin WristOx2 3150, Air Liquide Medical Systems, France) and recorded: before, at each stop and just before the end of the trial.

1.2.5 Efficiency

Athlete's efficiency (ϵ) during each 1500 m can be approximated (without taking into account the phosphagens contribution) with the following equation:

$$\epsilon = \frac{\int_{t_0}^{t_f} P_{handle}(t)\, dt}{E_{oxi} + E_{non-oxi}} \tag{S5}$$

This efficiency includes the metabolic and technical efficiency of each athlete.

1.3 Psychological variables

Participants indicated that they were recovered from the warm-up and ready to perform optimally in the upcoming trial. The baseline RPE was thus considered as 1/10. Rating of perceived exertion (RPE) using

the Borg CR10 scale (Borg, 1982) was indicated by the participants during the stop after each 500 m and at the end of the 1500 m. Area under the RPE curve (AUC_{RPE}) for each condition was calculated (similarly to AUC_{La}). After the three trials, each participant ranked the trials according to their preference (from 3 = most favorite to 1 = least favorite).

2 STATISTICAL RESULTS OF COMPARISONS

The paired-Wilcoxon rank test was then used to compare the groups two by two. The statistical significance threshold was set at p-value < 0.05. The effect size was evaluated through a rank-biserial correlation. Numerical values of p-values and rank-biserial correlation are available in the attached data sheet.

REFERENCES

Borg, G. A. (1982). Psychophysical bases of perceived exertion. *Medicine and Science in Sports and Exercise* 14, 377–381

Boyas, S., Nordez, A., Cornu, C., and Guével, A. (2006). Power responses of a rowing ergometer: Mechanical sensors vs. concept2® measurement system. *International Journal of Sports Medicine* 27, 830–833. doi:10.1055/s-2006-923774

Di Prampero, P. E. (1981). Energetics of muscular exercise. In *Reviews of Physiology, Biochemistry and Pharmacology, Volume 89* (Springer Berlin Heidelberg), vol. 89. 143–222. doi:10.1007/BFb0035266. Series Title: Reviews of Physiology, Biochemistry and Pharmacology

Geyssant, A., Dormois, D., Barthelemy, J. C., and Lacour, J. R. (1985). Lactate determination with the lactate analyser LA 640: a critical study. *Scandinavian Journal of Clinical and Laboratory Investigation* 45, 145–149. doi:10.3109/00365518509160987

Ingham, S. A., Carter, H., Whyte, G. P., and Doust, J. H. (2007). Comparison of the oxygen uptake kinetics of club and olympic champion rowers. *Medicine & Science in Sports & Exercise* 39, 865–871. doi:10.1249/mss.0b013e31803350c7

Messonnier, L., Freund, H., Bourdin, M., Belli, A., and Lacour, J.-R. (1997). Lactate exchange and removal abilities in rowing performance:. *Medicine &amp Science in Sports &amp Exercise* 29, 396–401. doi:10.1097/00005768-199703000-00016
